# Q&A: Robotics as a tool to understand the brain

**DOI:** 10.1186/1741-7007-8-92

**Published:** 2010-07-23

**Authors:** Daniel M Wolpert, J Randall Flanagan

**Affiliations:** 1 Department of Engineering, University of Cambridge, Trumpington Street, Cambridge CB2 1PZ, UK; 2 Department of Psychology and Centre for Neuroscience Studies, Queen's University, Kingston, ON K7L 3N6, Canada

## What type of robots are we talking about?

Although humanoid robots are often in the press, most robotic devices found in neuroscience labs around the world are specialized devices for controlling stimuli and creating virtual environments. Most robots consist of a series of links that allow the end of the robotic interface to move either in a two-dimensional plane or three-dimensional space, and look more like a fancy Anglepoise lamp than a human. The configuration of the robot is tracked with sensors at a high rate and computer-controlled motors can change the configuration of the robot. In this way the neuroscientist can control the position of the robot and the forces applied by the robotic interface.

## What can these robots do?

**Figure 1:**
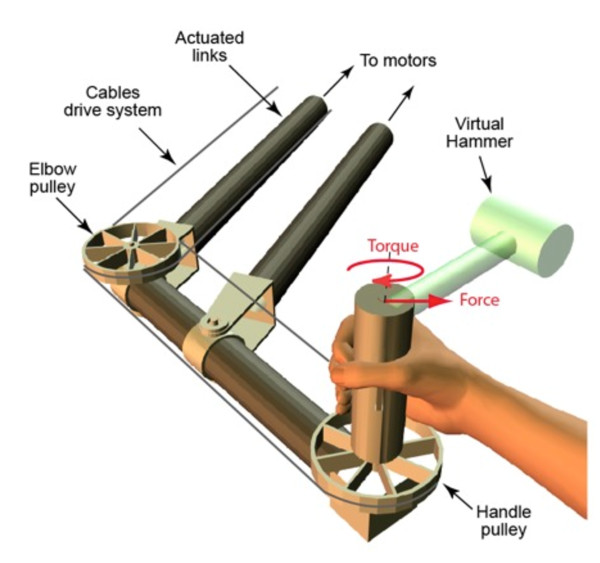
**A robot used in a recent experiment on motor control**. The schematic shows a Wrist-bot being used to simulate a virtual hammer manipulated in the horizontal plane. The robotic interface consists of a linked structure actuated by two motors (not shown) that can translate the handle in the horizontal plane. In addition a third motor drives a cable system to rotate the handle. In this way both the forces and torques at the handle can be controlled depending on the handle's position and orientation (and higher time derivatives) to simulate arbitrary dynamics - in this case a virtual hammer is simulated. Modified from *Current Biology*, Vol. 20, Ingram *et al*., Multiple grasp-specific representations of tool dynamics mediate skillful manipulation, Copyright (2010), with permission from Elsevier.

Robots have been particularly important in areas of neuroscience that focus on physical interactions with the world, including haptics (the study of touch) and sensorimotor control (the study of movement). Indeed, robots have done for these areas what computer monitors have done for visual neuroscience. For decades, visual neuroscientists had a substantial advantage because generating visual stimuli is straightforward using computers and monitors. This allowed the precise experimental control over visual inputs necessary to test between hypotheses in visual neuroscience. However, when it came to haptics and sensorimotor control, it has been far harder to control the stimuli. For example, to study haptics one might want to create arbitrary physical objects for tactile exploration, whereas to study motor learning one might want to generate physical objects that have novel dynamical properties and change these properties in real time. Robotic interfaces allow precisely this type of manipulation. In many motor control experiments, the participant holds and moves the end of a robotic interface (Figure 1) and the forces delivered by the robot to the participant's hand depend on the hand's position and velocity (the hand's state). The mapping between the hand's state and the forces applied by the robot is computer controlled and, within the capabilities of the robots, the type of mapping is only limited by the experimenter's imagination.

## What sorts of things can these robots simulate?

Although the mapping between state and force is arbitrary, in practice, experiments tend to fall into several distinct types. In many studies of haptic exploration, the robotic interface is used to simulate static objects such as a sphere. This can be achieved by simulating the surface of the object as a stiff spring that generates forces perpendicular to the surface. In this way, the harder you try to push into the surface the more the stiff spring is extended and the larger the resistive force. In motor control studies, robots are often used to simulate physical objects that move when force is applied and therefore have dynamics. Although it is possible to construct real objects with different shapes, surface compliance and dynamics, this is often a painstaking process and is limited in flexibility. By using robots, objects with a wide range of properties can be rendered and changed in real time. Moreover, it is possible to create objects with unusual properties, which are especially useful for studies of learning. For example, numerous studies of motor learning have used objects that, when moved in a horizontal plane, generate forces proportional to hand speed and directed orthogonal to the current direction of hand movement. These objects, referred to as viscous curl force-fields (so-called because one can plot the force vectors as a function of the state - in this case velocity of the hand), have allowed neuroscientists to study motor learning in highly novel situations unlikely to have been experienced outside the lab. In addition, robotic interfaces can be used to constrain movements of the hand to particular paths through space, apply force pulses and perturbations to the arm during movement, and move a person's arm passively around the workspace.

## Which fields in neuroscience have been advanced by the use of robotics?

Four fields have been substantially aided by the use of robotic interfaces. The study of haptics aims to understand how tactile and other somatosensory inputs are processed. Second, the field of sensorimotor control focuses on how the brain controls movements of both the body and objects the body interacts with, and how we learn new motor skills. Third, the field of rehabilitation therapy aims to understand how to facilitate recovery from various insults to the system such as stroke or spinal cord injury. Finally, the burgeoning field of brain-machine interfaces seeks to develop techniques by which the brain can directly control external devices.

## Can you give some examples of how robotics has advanced haptics research?

When you run your finger over a horizontal surface and feel a bump, what processes lead to the perception of the bump? It could be that perception of the bump is based on the position of the finger, which rises and then falls as it goes over the bump. Alternatively, perception of the bump could be based on horizontal force acting on the finger that resists and then assists lateral motion of the finger as it goes up and then down the bump, respectively. Testing these hypotheses was achieved using a simple robotic interface that could simulate horizontal forces to resist and then assist the finger, as though it were going over a bump, critically while maintaining the finger in the horizontal plane at all times. Surprisingly, the percept was that of a bump, indicating that the force cues were important.

Another key question is how haptic information is combined with other information such as visual inputs to form a single percept. When we pick up a viewed object, we receive both haptic and visual information about its width. An optimal estimator, which aims to estimate the object's width with the smallest error (that is, optimally), would combine these two sources of information using a weighted average where the weighting of each source depends on its reliability. By attaching the ends of two lightweight robots to the tip of the index finger and thumb, it is possible to simulate objects and control the width experienced haptically. By combining this robotic setup with a virtual-reality visual display, a recent study created objects with different haptic and visual widths and was able to determine the weighting given to each source of information in the perception of width, weightings that matched the predictions of an optimal estimator.

To examine the first stages of tactile processing, very fine control over the tactile input is required. By developing a robotic interface that can precisely control the position and orientation of a tactile probe over time, and combining tactile stimulation with microneurography (recordings from tactile afferent nerves), it has been possible to show in humans that the timing of first spikes from tactile primary afferents carry a great deal of information about properties of the object being contacted, such as curvature and friction.

## How has the study of sensorimotor control been advanced?

There are two areas in which robotic interfaces have been of particular use - the study of motor learning and of stiffness control. To study motor learning it is important in the lab to present participants with tasks they have not seen outside the lab. Robots have been vital in enabling a range of new tasks to be studied. For example, studies of how people adapt their reaching movement when moving an object with unusual dynamics have led to an understanding of how dynamics are represented, how this representation changes on a trial-by-trial basis with experience, and how different tasks interfere with or facilitate each other. Robots have also been important for studying stiffness control, the ability to stiffen our limbs through muscle co-contraction in order to deal with unpredictable loads. Robots can be used to measure arm stiffness by rapidly shifting the position of the arm and measuring the restoring forces before reflexive mechanisms are activated that influence the muscle forces. By perturbing the hand in different directions, it is possible to build up a picture of the stiffness of the human arm in different directions. Such studies have shown that people control their stiffness in a complex way and can tune their stiffness, although to a limited extent, so as to match their stiffness optimally to the task at hand.

## How has the study of rehabilitation therapy been advanced?

Much of the work done by physiotherapists in rehabilitation involves direct physical interactions between the therapist and the patient that are difficult to quantify precisely. This can make it challenging to test between different therapies. Several research groups are currently assessing whether robotic systems can be used for rehabilitation. The basic idea is that the patient is attached to a robot that can partially assist the patient's movements. As the patient improves, the contribution of the robot can be decreased. The patient is encouraged to play a range of movement games. Results from such studies, which can be quantified both by the robotic interface and standard tests, are encouraging, and it is likely that we will see an increasing involvement of such devices in the clinic. In the future, robots will probably be an important tool for physiotherapists that will enable them to quantify performance and design tailor-made robotic therapies.

## How has the study of brain-machine interfaces been advanced?

Over the past few years there has been substantial interest in trying to extract meaningful information from signals recorded from the brain to control external devices. The main driving force for this research is to develop devices that will allow patients with neural impairments, including spinal cord injury and motor neuron disease, as well as amputees to effect movement. The idea is to record the pattern of activity of neurons in brain areas such as motor cortex, and use this activity to either drive the muscles directly or control a robotic device. In addition to such medical uses, the military also has an interest in allowing normal brains to directly control hardware. Several groups have now graduated from using neural signals to driving cursor movement on a screen to using the signals to drive a robotic system, with some groups using implanted arrays in nonhuman primate cortex to control a robot that the animal uses to feed itself. At present, such systems do not fully close the loop; while the animal can see the robotic interface, and therefore guide it visually, effective tactile feedback, which may allow a finer manipulation ability, has yet to be fully developed.

## What type of commercial robots are there?

To our knowledge the first robotic interface that had a major impact on sensorimotor neuroscience was developed in the 1980s by Neville Hogan's group at the Massachusetts Institute of Technology. They developed a planar two-degree-of-freedom manipulandum with a handle that subjects could hold and move around and that could perturb the hand during reaching movements. Since then, several extensions of this basic design have reached the market. A device that has been particularly popular for haptic research is the Phantom Haptic Interface developed by SensAble Technologies. Although limited in the forces it can generate, this device is very lightweight and its endpoint can be positioned and oriented in three-dimensional space. Whereas these systems apply forces to the hand, one device has been developed especially to apply torques directly to the segments of the arm. This exoskeletal device, called the Kinarm, allows precise control over the torques delivered to individual joints, allowing more control over the types of perturbations that can be investigated. In addition, there is a range of more anthropomorphic robots, such as the Sarcos system, that are used as test beds for hypotheses about the way that humans control the body.

## What does the future hold for the use of robots in neuroscience?

Today, robots are where computers were 30 years ago. That is, they are highly specialized and expensive devices found in a handful of labs around the world and require considerable expertise to use. However, we expect that in the years ahead, robots will become affordable, flexible and easy to use and that many labs will employ a range of robotic devices for neuroscience experiments and as a theoretical test bed.

## Where can I find out more? (final Q&A question)

Articles

Atkeson CG, Hale J, Pollick F, Riley M, Kotosaka S, Schaal S, Shibata T, Tevatia G, Vijayakumar S, Ude A, Kawato M: **Using humanoid robots to study human behavior**. *IEEE Intelligent Systems: Special Issue on Humanoid Robotics *2000, **15**, 46-56.

Burdet E, Osu R, Franklin D, Milner T, Kawato M: **The central nervous system stabilizes unstable dynamics by learning optimal impedance**. *Nature *2001, **414:**446-449.

Ernst MO, Banks M: **Humans integrate visual and haptic information in a statistically optimal fashion**. *Nature *2002, **415:**429-433.

Howard I, Ingram J, Wolpert D: **A modular planar robotic manipulandum with end-point torque control**. *J Neurosci Methods *2009, **181:**199-211.

Ingram JN, Howard IS, Flanagan JR, Wolpert DM: **Multiple grasp-specific representations of tool dynamics mediate skillful manipulation**. *Curr Biol *2010, **20:**618-623.

Johansson RS, Birznieks I: **First spikes in ensembles of human tactile afferents code complex spatial fingertip events**. *Nat Neurosci *2004, **7:**170-177.

Lackner J, DiZio P: **Motor control and learning in altered dynamic environments**. *Curr Opin Neurobiol *2005, **15:**653-639.

Robles-De-La-Torre G, Hayward V: **Force can overcome object geometry in the perception of shape through active touch**. *Nature *2001, **412:**445-448.

Schaal S, Schweighofer N: **Computational motor control in humans and robots**. *Curr Opin Neurobiol *2005, **15:**675-682.

Scott S: **Apparatus for measuring and perturbing shoulder and elbow joint positions and torques during reaching**. *J Neurosci Methods *1999, **89:**119-127.

Shadmehr R, Mussa-Ivaldi FA: **Adaptive representation of dynamics during learning of a motor task**. *J Neurosci *1994, **14:**3208-3224.

Velliste M, Perel S, Spalding MC, Whitford AS, Schwartz AB: **Cortical control of a prosthetic arm for self-feeding**. *Nature *2008, **453:**1098-1101.

Volpe BT, Ferraro M, Lynch D, Christos P, Krol J, Trudell C, Krebs HI, Hogan N: **Robotics and other devices in the treatment of patients recovering from stroke**. *Curr Neurol Neurosci Rep *2005, **5:**465-470.

Volpe BT, Huerta PT, Zipse JL, Rykman A, Edwards D, Dipietro L, Hogan N, Krebs HI: **Robotic devices as therapeutic and diagnostic tools for stroke recovery**. *Arch Neurol *2009, **66:**1086-1090.

